# High Incidence of Hypoglycemia from an Audit of Glycemic Control and Management in Patients with Diabetes in a Cardiac Unit

**DOI:** 10.3389/fendo.2013.00168

**Published:** 2013-11-07

**Authors:** May Lea Ong, Helena J. Teede, Sophia Zoungas, Jennifer L. A. Wong

**Affiliations:** ^1^Monash Health, Clayton, VIC, Australia; ^2^Monash University, Clayton, VIC, Australia; ^3^School of Public Health and Preventive Medicine, Clayton, VIC, Australia

**Keywords:** diabetes, hypoglycemia, glycemia, cardiac, coronary care

## Introduction

The coronary care unit (CCU) model of care has evolved considerably over the past several decades and emerged as one of the most important advances in the care of patients with acute coronary syndrome (ACS). The concept of a specific unit for patients with ACS equipped with highly trained and specialized staff was initially introduced in the 1960s, but it was not until 1980s that the idea took off with the advent of coronary angiography and fibrinolytic therapy (1). However, in this new modern environment with high rate of cardiac interventions and patient turnover, many challenges have been created with competing demands for nursing time. These challenges are even greater in patients with diabetes frequently who require rapid intensification of therapy to optimize blood glucose levels whilst needing procedures which require fasting.

The prevalence of diabetes in hospitalized patients is significant and is on an increasing trend. However, this figure is not well documented and hence significantly underestimated (2, 3). More than a decade ago, 12.4% of hospital discharges in the United States have diabetes documented as a diagnosis (4). More recently, the prevalence of diabetes was recorded from at least 10% and up to 25% of adult patients admitted to hospitals in the United Kingdom (5). Not surprisingly, this figure is increased in high-risk groups, particularly, patients admitted to a CCU. A 11-years review of patients admitted to a CCU in Italy found a prevalence of diabetes at 31.5% and almost 40% with concomitant ST-elevation myocardial infarction (6).

The combination of the busy modern CCU environment and the high and increasing number of patients with diabetes creates a high-risk setting for hypoglycemia. Severe hypoglycemia is known to be associated with increased morbidity and mortality in hospitalized patients and has been postulated to be associated to a range of adverse clinical outcomes or at least a marker of vulnerability to such events (7). This finding has been extended to patients hospitalized with ACS, where severe hypoglycemia was a major predictor of cardiovascular death (8). Similarly, hypoglycemia has also been implicated in the excess all-cause mortality observed in this patient group (9).

## Methods and Results

In the setting of the overwhelming concern about the negative association between hypoglycemia and adverse cardiovascular events, we undertook a prospective audit of glycemic control and management of 46 patients with diabetes in the cardiac unit of one of the largest tertiary health center in Australia, Monash Health (MH) during May and June 2012. The characteristics of the patients, length of stay and the principle diagnosis on admission are presented on the Table 1.

**Table 1:** **Baseline characteristics of patients, length of stay, principal diagnoses, glycemic management and control**.

**Patient characteristic**	
Mean age ± SD years	69 ± 10
Male, *n* (%)	37 (80)
Female, *n* (%)	9 (20)
Duration of diabetes ± SD years	12 ± 10
Mean HbA1c on admission ± SD	7.3 ± 1.5
**Length of stay ± SD days**	8 ± 5
**Principal diagnoses, *n* (%)**
Acute coronary syndrome	25 (54)
Cardiac failure	10 (22)
Arrhythmia	3 (7)
Conduction defect	3 (7)
Chest pain for investigation	2 (4)
Valvular condition	2 (4)
**Outpatient diabetes management, *n* (%)**
OHA	28 (61)
Insulin	6 (13)
OHA and insulin	7 (15)
**Inpatient diabetes management, *n* (%)**
Same as outpatient	20 (43)
Additional supplemental insulin	21 (46)
Additional pre-mixed insulin	3 (7)
Additional basal insulin	2 (4)
Insulin infusion	1 (2)
**Inpatient glycemic control**
Normoglycemic readings (4–10 mmol/l), *n* (%)	985 (58)
Hyperglycemia readings (>10.1 mmol/l), *n* (%)	554 (33)
Mild hypoglycemia readings (≥2.8–3.9 mmol/l), *n* (%)	40 (2)
Severe hypoglycemia reading (<2.8 mmol/l) *n* (%)	8 (20)
Hypoglycemic patients, *n* (%)	15 (33)
Recurrence, *n* (%)	11 (73)
Severe, *n* (%)	5 (33)

SD, standard deviation.

On admission the majority of the patients were treated with oral glucose lowering agents alone (61%) and these agents were continued during hospitalization in 43% of patient, with only 46% of patients receiving additional supplemental insulin. The majority of blood glucose measurements were normoglycemic (58%). In 33% of patients, at least one hypoglycemic episode was recorded, with the majority having a recurrence (73%). However, most hypoglycemic episodes were mild (80%).

## Conclusion

In light of these findings, barriers to the prevention of hypoglycemia were explored with staff, which included competing demands on nurses resulting in variable administration times for glucose lowering medications and fasting for procedures with repeated rescheduling. Identifying those at risk and intervening before and after hypoglycemia occurs were clearly important given the high rates of recurrence.

These findings created an impetus for strategies to prevent hypoglycemia especially in the modern CCU environment. In the case of MH, strategies implemented after the audit included in-service training highlighting the need to prioritize those with diabetes on procedural lists, engagement of a diabetes nurse educators to support CCU nursing staff and implementation of hospital policies concerning prevention and management of hypoglycemia in fasting patients with diabetes. The MH policy for pre-procedural medication management in fasting patients with diabetes was also established and involved omitting morning dose of oral hypoglycemics, short-acting, and pre-mixed insulin on the day of the procedure when fasting from midnight. However, if fasting from breakfast, oral hypoglycemics will still need to be omitted, whereas if on insulin, only 80 and 50% of the usual dose should be administered if on short-acting and pre-mixed insulin respectively.

## Key Messages

- Coronary care unit creates a high-risk setting for hypoglycemia which is known to be associated with increased mortality and morbidity
- Patients with an initial episode of hypoglycemia are likely to have a recurrence
- Proactive strategies need to be in place to reduce the risk of hypoglycemia
